# Knowledge Gaps, Sleep Disturbances, and Energy Imbalance Among Female Field Hockey Players

**DOI:** 10.3390/nu17243934

**Published:** 2025-12-16

**Authors:** Xavier Puchalt-Urbano, Andrea Calderón-García, Jesús R. Huertas, Antonio Jesús Sánchez-Oliver, Cristina López de la Torre, Elena Aguila-Aguilar, Pablo Jesús Lopez Soto, Raúl M. Luque, Fernando Mata-Ordóñez

**Affiliations:** 1 Department of Pharmacy and Nutrition, Faculty of Biomedical and Health Sciences, Universidad Europea de Madrid, 28670 Madrid, Spain; 2 Institutes of Nutrition and Food Technology (INYTA), Biomedical Research Centre “José Mataix”, University of Granada, 18071 Granada, Spain; 3 Department of Physiology, University of Granada, 18071 Granada, Spain; 4 Departamento de Motricidad Humana y Rendimiento Deportivo, Universidad de Sevilla, 41013 Sevilla, Spain; 5 Department of Biomedicine and Dentistry, Faculty of Biomedical Sciences and Sports, Universidad Europea de Andalucía, 29010 Málaga, Spain; 6 Departamento de Enfermería, Farmacología y Fisioterapia, Universidad de Córdoba, 14071 Córdoba, Spain; 7 Instituto Maimónides de Investigación Biomédica de Córdoba (IMIBIC), Hospital Universitario Reina Sofía (HURS), 14004 Córdoba, Spain; 8 Departamento de Biología Celular, Fisiología e Inmunología, Universidad de Córdoba, 14014 Córdoba, Spain; 9 Centro de Investigación Biomédica en Red de Fisiopatología de la Obesidad y Nutrición, (CIBERobn), 28019 Madrid, Spain

**Keywords:** field hockey, low energy availability, sports nutrition, sports supplements, sleep

## Abstract

**Background:** Field hockey is a high-intensity intermittent sport, where nutrition and sleep play an important role in the performance and health of all players, especially in women, who often exhibit a dietary pattern characterized by low energy and carbohydrate intake, along with poor nighttime habits. The purpose of this study is to evaluate the profile of female field hockey players by analyzing their energy and macronutrient intake, prevalence of LEA risk, use of SS, and sleep characteristics. **Methods:** A cross-sectional, observational, and descriptive study was carried out with 75 female players. Validated questionnaires were used to determine general sports nutrition knowledge (NUKYA) and specific knowledge of carbohydrates in sports (CEAC-Q), sleep quality (ASSQ), low energy availability risk (LEAF-Q), and use of sport supplements. In addition, a 7-day dietary record was analyzed. **Results:** Players showed a high general nutrition knowledge (NUKYA mean: 66.0 ± 8.5 points; 68.0% scored high), but a pronounced deficiency in specific carbohydrate knowledge (CEAC-Q mean: 24.3 ± 14.9 points; 84.0% scored low). Dietary intake analysis revealed significant deficiencies: mean energy intake was 31.9 ± 10.8 kcal/kg/day, resulting in insufficient intake for 78.7% of players. Carbohydrate intake was particularly low (3.6 ± 1.5 g/kg/day), with 86.7% failing to meet recommendations. Furthermore, 33.3% of players were at risk of Low Energy Availability (LEA). LEA risk (LEAF-Q score) was negatively correlated with both protein (r = −0.363; *p* = 0.001) and carbohydrate intake (r = −0.347; *p* = 0.003). Regarding sleep, the mean disturbance score (SDS) was 6.5 ± 2.9 (mild disturbance), with 33.3% showing moderate–severe disturbance, and 92.0% sleeping ≤8 h. Finally, 78.7% of players used supplements, with usage correlated with CEAC-Q scores (r = 0.233; *p* = 0.044), and 86.4% were guided by non-professional sources. **Conclusions:** Female hockey players do not meet dietary recommendations for energy and carbohydrates and exhibit a high prevalence of low energy availability and sleep disturbances, despite having acceptable general sports nutrition knowledge. It is recommended to implement specific educational and nutritional strategies to enhance the knowledge, performance, and health of female hockey players.

## 1. Introduction

Field hockey is a team sport in which teams of ten players each, plus a goalkeeper, compete against each other. Players are categorized as forwards, midfielders, and defenders. The most recent rules stipulate that matches are played in four quarters of fifteen minutes, with two minutes of rest between the first and second and the third and fourth quarters, and five minutes of rest between second and third quarters [1]. It is categorized as a high-intensity, intermittent sport with matches of short duration but with a high volume of play [2,3,4,5]. Positional roles in field hockey impose markedly different physical and physiological demands: Defenders typically cover greater total distance at lower relative intensity, midfielders accumulate the highest moderate-intensity workload due to their transitional role, and forwards perform repeated high-intensity sprints and reach higher percentages of maximal velocity [3]. It also has a higher injury rate than other team sports [6]. This highlights the importance of adequate nutrition and optimal rest for the performance and health of field hockey players [7,8,9,10,11], especially for female players, whose hormonal profile is more complex and variable [12].

The major nutritional concern among female athletes is low energy availability (LEA), a state in which dietary energy intake is insufficient to cover exercise energy expenditure and maintain optimal physiological functions, affecting between 23% and 79% of women athletes across various sports [7]. Although field hockey is not traditionally classified as an aesthetic or weight-sensitive discipline, several factors may predispose players to LEA. First, the sport involves high-intensity intermittent efforts and prolonged training sessions, which markedly increase energy expenditure; if players fail to adjust their intake accordingly, unintentional LEA can occur [13]. Second, cultural and performance-related pressures—such as the belief that reducing body mass will improve speed and agility—may lead some athletes to restrict energy intake, despite the importance of strength and endurance for performance [7]. Finally, emerging evidence highlights that LEA often arises inadvertently during congested competition schedules or periods of intensified training, when logistical constraints and inadequate nutritional planning limit opportunities for sufficient fueling [13]. These scenarios underscore the need for education and monitoring strategies to prevent problematic LEA and its associated health and performance consequences in field hockey athletes. In addition, it is reported that between 39.0% and 78.0% of athletes have sleep problems [11,14], which may aggravate LEA effects due to their physiological restorative effects [10].

Sleep is increasingly recognized as a critical determinant of athletic performance, recovery, and overall health. Adequate sleep supports physiological restoration, cognitive function, immune response, and hormonal regulation, all of which are essential for athletes exposed to high physical and psychological stress during training and competition [9,11]. Evidence suggests that elite athletes often experience sleep inadequacies, characterized by short duration, poor quality, and irregular patterns, which can impair recovery and increase the risk of illness and injury [11]. Female athletes may be particularly vulnerable due to hormonal fluctuations and sociocultural factors influencing sleep behaviors [14]. In team sports such as field hockey, late training sessions, travel demands, and pre-competition anxiety further exacerbate sleep disturbances, potentially compromising performance and well-being [10]. Therefore, examining sleep characteristics in female field hockey players is essential to identify specific challenges and develop targeted strategies to optimize recovery and performance.

In the field of sports nutrition, ensuring adequate nutrient intake through diet is established as a fundamental priority. Although obtaining certain compounds in sufficient quantities to avoid deficits or achieve ergogenic effects can be complicated, sports supplements (SS) can be a useful tool for supplementing the diet and optimizing performance [15]. SS are widely consumed by athletes, even though they sometimes lack adequate knowledge about their effects and methods of use [16]. However, the main reason for using them is to improve athletic performance [16,17]; some studies [18,19] report that SS knowledge is low. Another aspect that affects LEA risk is general nutrition knowledge [19,20] as it may determine food intake [20,21,22], even though female athletes do not have adequate energy and nutrient intake [23], in addition to inappropriate SS use [24] and, overall, low scores on questionnaires assessing nutrition knowledge [25]. Some field hockey research [25,26] reveals important challenges in nutrition. In the first place, even though many field hockey players show interest in learning about nutrition, their knowledge is low, especially among female field hockey players. In addition, there is a clear difficulty in translating this knowledge into appropriate food choices [26]. As for the consumption of SS, Muzaffar Ali Khan Khattak [26] observed that, among female field hockey players, there is a preference for the use of vitamins and minerals, and the main motivation for using SS is the treatment of injuries.

It should be noted that one of the main limitations of women’s sport nutrition research is that female athletes are underrepresented in scientific publications covering athletic performance [27,28], especially in research addressing the nutrient needs of female athletes and supplementation [29,30,31]. This is especially noticeable in field hockey, as there are no studies focusing on the previously described variables in female hockey players.

Therefore, the objective of this study is to evaluate the profile of female field hockey players by analyzing their energy and macronutrient intake, prevalence of LEA risk, use of SS, and sleep characteristics. Based on previous evidence of inadequate dietary practices and limited nutrition knowledge in female athletes, we hypothesize that a substantial proportion of players will present insufficient energy intake relative to their training demands, a high prevalence of LEA risk, suboptimal sleep quality, and patterns of SS use that may not align with evidence-based recommendations.

## 2. Materials and Methods

### 2.1. Study Design

**Figure 1:**
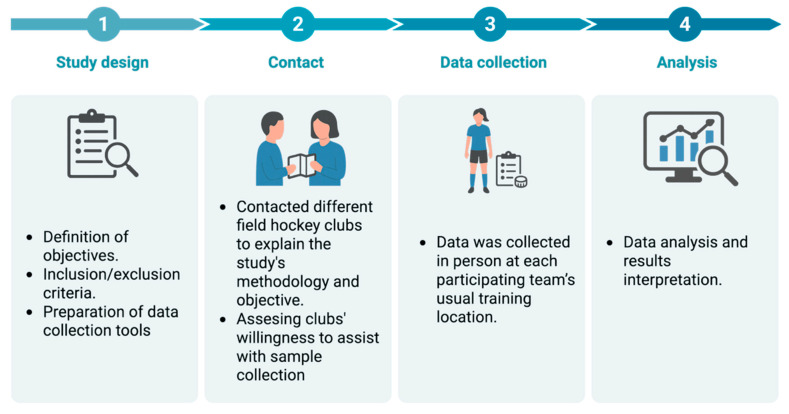
Study phases and workflow. Created in BioRender. Mata Ordóñez, F. (2025) https://BioRender.com/ys3nlqa (accessed on 1 February 2025).

To establish the dietary and sleep habits profile of female field hockey players, a non-experimental, cross-sectional, and descriptive study was conducted. Participant recruitment was carried out through the Valencian Community Hockey Federation and several field hockey clubs in the region. A non-probabilistic sampling method was used, and the final sample consisted of female field hockey players competing in the Honor B Division League. The study was conducted in several stages to ensure the quality and feasibility of data collection (Figure 1).

### 2.2. Participants and Procedure

The method of recruiting participants was initially carried out by contacting clubs through the Hockey Federation, sending detailed information about the objectives and methodology of the study via email. Subsequently, data collection was carried out in person at each team’s usual training venue, which minimized disruption to the players’ training routine and ensured a comfortable and familiar environment for completing questionnaires.

The sample size was determined using the Australian Bureau of Statistics (https://www.abs.gov.au/websitedbs/D3310114.nsf/home/Sample+Size+Calculator, accessed on 1 February 2025) Sample Size Calculator, with a 95% confidence level. The target population consisted of 256 female field hockey players belonging to the Honor B Division League. Assuming an expected proportion of 0.50, the final sample included 75 players, with a confidence interval of 0.09, a standard error of 0.05, and a relative standard error of 9.79. Consequently, all participants competed in the Honor B Division League, which corresponds to the second national division of field hockey in Spain.

The inclusion criteria to participate in this study were as follows: being a female field hockey player; holding a valid federation license in field hockey; being in the competitive phase of the season; being between 18 and 35 years old; and meeting at least Tier 3 of the Sport Performance Classification Framework [32]. Exclusion criteria included not meeting any of the inclusion criteria. In addition, athletes presenting any medical condition affecting metabolism, hormonal status, or dietary intake; those undergoing rehabilitation for an injury that prevented them from training normally; those who had used medication during the last trimester; and those who were pregnant or breastfeeding were also excluded from the study.

### 2.3. Instruments

A total of six questionnaires were used to evaluate nutrition knowledge [33,34], subjective sleep assessment [35], use of SS [36], and LEA risk [37], and prior to completion, they were explained.

#### 2.3.1. Nutrition Knowledge Questionnaire

A total of two questionnaires were used to assess nutrition knowledge: The Questionnaire on Nutrition Knowledge for Young and Adult Athletes (NUKYA) [33] was used to determine general knowledge in the female athlete participants; and the Carbohydrate for Endurance Athletes in Competition Questionnaire (CEAC-Q) [34] was used to establish specific sport nutrition knowledge. Both questionnaires contain multiple-choice questions and only one correct answer.

The NUKYA questionnaire [33] has 59 questions, grouped into four sections: (1) macronutrients (49.1 points), (2) micronutrients (32.3 points), (3) hydration (13.6 points), and (4) periodization (5.1). It is a validated and reliable questionnaire (Cronbach α = 0.849, test–retest reliability r = 0.895) for use in athletes. To obtain the questionnaire score, it was established that correct answers would earn one point (+1 point), while incorrect answers or answers marked as ‘I do not know’ would earn no points (0 points), with a maximum score of 100 points. The scores were classified as follows: (1) very low (0 to 19 points), (2) low (20 to 39 points), (3) medium (40 to 59 points), (4) high (60 to 79 points), and (5) very high (80 to 100 points).

The CEAC-Q questionnaire [34] is a validated (Cronbach α = 0,82) and useful (test–retest reliability r = 0.742) instrument, with 25 questions divided into five sections, each worth 20 points, and a maximum score of 100 points: (1) carbohydrate storage and metabolism, (2) pre-competition carbohydrate loading, (3) pre-competition carbohydrate meal, (4) carbohydrate during competition, and (5) carbohydrate for post-competition recovery. The CEAC-Q scores were classified as follows: (1) low (0 to 39 points), (2) medium (40 to 69 points), and (3) high (70 to 100 points).

#### 2.3.2. Sleep Assessment

The Athlete Sleep Screening Questionnaire (ASSQ) [35] was used for the subjective assessment of sleep in athletes, as it is validated and useful (Cronbach α = 0.74 y test–retest reliability r = 0.86) [35,38], and it is the only and specific questionnaire for the subjective assessment of sleep in athletes [11]. The ASSQ is used to identify sleep disturbances and daytime dysfunction in order to offer interventions based on the type and severity of the problem detected in athletes [38]. This questionnaire is divided into six different areas of sleep assessment: (1) total duration, (2) characteristics of insomnia, (3) quality, (4) chronotype, (5) nocturnal breathing disturbances, and (6) travel problems. To evaluate the questionnaire, the sleep problem score (SDS) must be calculated, which ranges from 0 to 17 points and is interpreted as follows: (1) no sleep disturbances for those who obtained 0 to 4 points, (2) mild disturbances for scores of 5 to 7 points, (3) moderate disturbance for 8 to 10 points, and (4) severe disturbance for 11 to 17 points [35,38].

Detailed frequency data for responses to the ASSQ, including items related to sleep duration, satisfaction, sleep onset latency, disturbances, use of medication, chronotype, preferred bedtime, travel-related sleep issues, snoring, breathing problems, caffeine intake, and electronic device use before bedtime, are provided as Supplementary Materials in Tables S7–S20.

#### 2.3.3. Sports Supplement Consumption Assessment

The use of SS was analyzed using a validated questionnaire [36], previously used in other studies [39,40,41,42,43,44], and which, according to a meta-analysis conducted by Knapik et al. [45], was one of the questionnaires selected as suitable, with a methodological score of 54% for obtaining adequate information on the use of supplements by athletes. The questionnaire is organized into three sections: (1) personal, anthropometric and demographic data, (2) contextualizing sport activity, and (3) information on SS use, the period of use (training period, competition period, competition and training period, transition period, always or never) and the time of use (before, during, of after training). To assess the use of SS, the 2019 Australian Institute of Sport (AIS) classification system [46] was used as a reference, which classifies supplements using the ABCD system. The system differentiates supplements into four different categories, according to their level of scientific evidence, safety of use, legality and effectiveness in improving athletic performance.

Detailed frequency data for categorical variables, including reasons and sources for supplement use, places of purchase, intake by time of day and timing, as well as consumption according to the AIS classification, are provided as Supplementary Materials in Tables S1–S6.

#### 2.3.4. Low Energy Availability Risk

The Low Energy Availability in Females Questionnaire (LEAF-Q) [37] was used to screen female athletes at risk of LEA. The LEAF-Q questionnaire contains 25 questions, grouped into different sections: (1) injuries, (2) gastrointestinal function, and (3) menstrual function and use of contraceptives. If the athlete scores more than 8 points, this indicates that she is at risk of LEA (42). The LEAF-Q questionnaire is a validated and useful tool with a sensitivity of 78% and specificity of 90% [37,38].

#### 2.3.5. Dietary Intake Assessment

Dietary intake was recorded using a 7-day dietary log, completed using Nutrium dietary software (Braga, Portugal, https://nutrium.com/, accessed on 1 February 2025) which is based on the Spanish Food Composition Database and the United States Department of Agriculture database. Each participant had their own personal and non-transferable username and password with which to record their food and liquid intake during the established period. In addition, prior to the end of the data collection session and due to the methodology of this study, all participants were instructed on how to use the application and correctly fill in the dietary diary.

The energy and macronutrient recommendations are defined in Table 1. Fiber intake was also studied, although there are specific recommendations of more than 25 g of fiber for the Spanish population [47], the recommendation chosen was more than 30 g per day, as this recommendation has greater health benefits for female athletes [48].

### 2.4. Statistical Analysis

Quantitative data have been expressed using the mean as a measure of central tendency and the standard deviation (SD) as a measure of dispersion, unless otherwise indicated. Normality was checked using the Shapiro–Wilk test, histograms, Q-Q plots, and box plots, and homoscedasticity was checked using Levenne’s test, with a confidence level of 95% and a *p*-value for significance of less than 0.05. The comparison of two independent groups was performed using the Mann–Whitney U test; for the comparison of several groups, the Kruskal–Wallis test was used with the Dunn test for post hoc contrast, or one-way analysis of variance together with the Bonferroni test for post hoc contrast, depending on the normality of the sample. Effect sizes (ES) were calculated using eta-squared (η^2^) for the comparison between playing positions, indicating small ES for 0.01, 0.06 for medium, and 0.014 for a large; for the comparison between two groups, Cohen’s d (d) in parametric comparisons, where 0.2, 0.5, and 0.8 indicated a small, moderate, and large ES, respectively, and the biserial correlation coefficient (r) was used in non-parametric comparisons, with an ES of 0.2 considered small, 0.5 moderate, and 0.8 large [53]. Correlations between variables were calculated using Pearson’s correlation coefficient, with correlation values ranging from 0.00 to 0.10 considered null, 0.10 to 0.39 slight, 0.50 to 0.69 moderate, 0.70 to 0.89 strong, and 0.90 to 1.00 very strong [54]. All analyses were conducted using the Statistical Package for the Social Sciences v.29.0 (IBM Corporation, Pittsburgh, PA, USA), and the visualizations were performed in RStudio (Posit Software, PBC, Boston, MA, USA) using R (R Core Team) and the ggplot 2 package v.4.0.1 (Springer-Verlag, New York, NY, USA).

## 3. Results

Table 2 shows demographics characteristics of the 75 female hockey players who decided to participate in this study.

### 3.1. Nutrition Knowledge

The average score for the NUKYA questionnaire was 66.0 ± 8.5 points, which was a higher average level for the participants, with no participants (0.0%) scoring low, 24.0% scoring medium, 68.0% scoring high, and 8.0% scoring very high.

**Figure 2:**
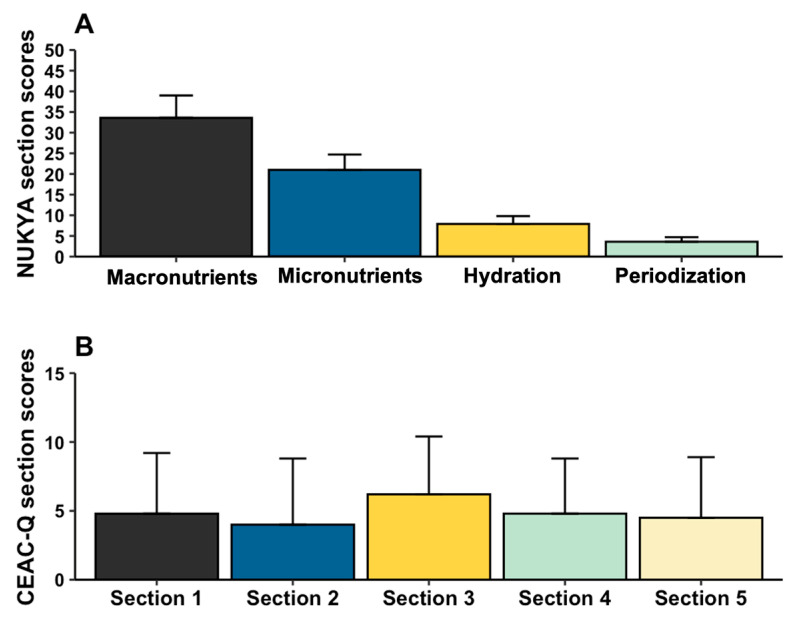
Section scores of NUKYA (**A**) and CEAC-Q (**B**) questionnaires. In (**B**), section 1 refers to carbohydrate storage and metabolism, section 2 to pre-competition carbohydrate loading, section 3 to before competition carbohydrate meal, section 4 to carbohydrate during competition, and section 5 refers to carbohydrate for post-competition recovery.

Figure 2A shows the average scores for each section of the NUKYA questionnaire. When broken down by accuracy rate, the periodization section (69.8 ± 22.4%) had the highest accuracy rate, followed by the macronutrient section (68.4 ± 11.1%); the third highest rate of correct answers was in the micronutrient section (65.3 ± 11.5%), and finally, the section with the lowest rate of correct answers was the hydration section (57.9 ± 13.9%).

For the CEAC-Q questionnaire, the mean score was 24.3 ± 14.9 points, demonstrating a low average level. In contrast to the NUKYA questionnaire, no hockey player participating in the study achieved a score classified as high (0.0%), while 16.0% were classified as having a moderate level of knowledge about carbohydrates, and the remaining 84.0% were classified as having a low level.

The scores for each section of the CEAC-Q questionnaire are shown in Figure 2B. The section with the highest success rate was section 3 with 31.2 ± 20.9%, followed by section 1 with 24.2 ± 21.2%, the third highest success rate was section 4 with 23.9 ± 20.3%, section 5 with 22.7 ± 21.9%, and finally, section 2 had the lowest success rate with 20 ± 24.0%.

**Figure 3:**
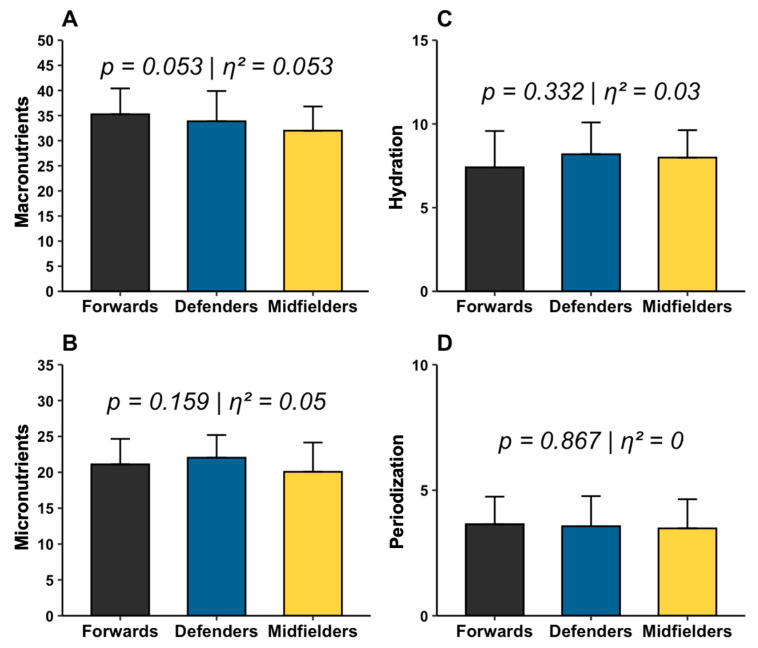
NUKYA questionnaire scores by playing position. Scores of NUKYA questionnaire: macronutrient section (**A**), micronutrient section (**B**), hydration section (**C**), and periodization section (**D**).

**Figure 4:**
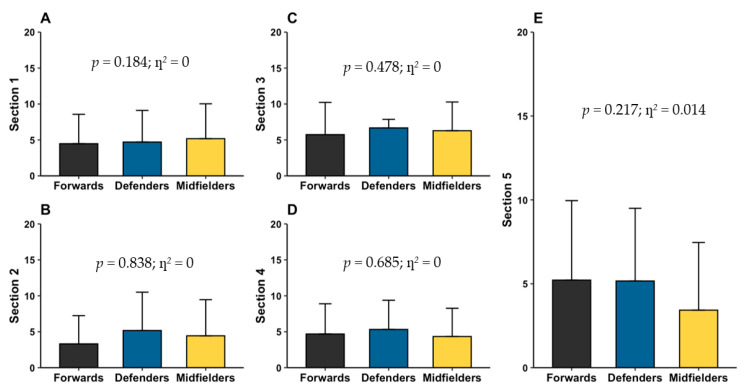
Scores of CEAC-Q questionnaires. Section 1 (**A**) refers to carbohydrate storage and metabolism, section 2 (**B**) to pre-competition carbohydrate loading, section 3 (**C**) pre-competition carbohydrate meals, section 4 (**D**) carbohydrate during competition, and section 5 (**E**) refers to carbohydrate for post-competition recovery.

The total and section scores of the NUKYA and CEAC-Q questionnaires by position are presented in Figure 3 and Figure 4, respectively. Analysis revealed that no significant differences were found between playing positions.

In terms of general sport nutrition knowledge levels, assessed with NUKYA questionnaire, defensive players obtained an average score of 67.7 ± 7.9 points, classified as a high level (0.0% very low and low, 12.5% medium, 79.2% high, and 8.3% very high), while forwards scored 67.4 ± 9.6 points, classified as high (0.0% very low and low, 21.7% medium, 65.2% high, and 13.0% very high), and finally, midfielders scored 63.5 ± 7.6 points, classified as high level (0.0% very low and low, 35.7% medium, 60.7% high, and 3.6% very high).

Regarding knowledge of carbohydrates, defensive players obtained an average score of 26.0 ± 15.2 points, classified as below average (79.2% low, 20.8% average, and 0% high), forwards scored 23.4 ±14.8 points, classified as low (82.6% low, 17.4% average, and 0% high), and finally, midfielders scored 23.7 ±15.2 points, classified as low level (89.3% low, 10.7% medium, and 0% high).

In the section concerning self-perceived knowledge of nutrition knowledge, 1.3% of players rated their knowledge as very high, 17.3% as high, 60.0% as medium, 17.3% as low, and 4.0% as very low. These results were compared with the actual scores obtained, revealing that 72.0% underestimated their level of nutrition knowledge (49.3% by one level below, 17.0% by two or more levels below), 21.3% had an accurate self-perception, and 6.7% overestimated their actual level of knowledge.

### 3.2. Subjective Sleep Evaluation

The mean SDS obtained through the ASSQ questionnaire was 6.5 ± 2.9 points, indicating a mild sleep disturbance, and the mean chronotype score was 7.1 ± 2.1 points, corresponding to a late-intermediate chronotype among all participating players (92.0% with a morning or intermediate chronotype and 8.0% with an evening chronotype). Overall, 25.3% presented no sleep disturbance, 41.3% had mild disturbance, 21.3% had moderate disturbance, and 12.0% had severe sleep disturbance. Table 3 shows the percentage distribution of SDS by playing position.

Across positions, mean SDS were 6.7 ± 3.0 for forwards, 6.3 ± 2.5 for defenders, and 6.4 ± 3.3 for midfielders, with no statistically significant differences between playing positions (*p* = 0.737; η^2^ = 0.000).

A total of 92.0% of respondents reported sleeping eight hours or less per night, 8.0% reported sleeping between eight and nine hours, and 0% slept more than nine hours on average in the preceding week. Regarding sleep satisfaction, 28.0% were dissatisfied with their nocturnal rest (1.3% very dissatisfied, 26.7% somewhat dissatisfied), 46.7% were satisfied (21.3% very satisfied, 25.3% somewhat satisfied), and the remaining 25.3% reported moderate satisfaction. Concerning difficulty falling asleep, 34.7% reported no problems, whereas 63.5% experienced such problems (49.3% one to two times per week, 10.7% three to four times per week, and 5.3% five to seven times per week).

Additionally, 90.7% of players reported using electronic devices during the hour before going to sleep (13.3% one to three times per week, 9.3% four to six times per week, and 68.0% every day). Table 4 presents the percentage distribution of responses for the sections on nocturnal sleep duration, sleep satisfaction, and difficulties in falling asleep.

### 3.3. Use of Sports Supplements

Of the 75 field hockey players surveyed, 78.7% reported using some type of SS, with a mean of 3.1 ± 2.2 supplements per person. The primary reason for SS use was to enhance sports performance (47.5%), followed by health maintenance (28.8%), improving physical appearance (8.5%), compensating for dietary deficiencies (6.8%), addressing health problems (3.4%), and, lastly, reasons such as necessity (1.7%), habit (1.7%), or obligation (1.7%).

The main sources of recommendation for SS use were friends (18.6%), the internet (15.3%), dietitians-nutritionists (13.6%), and coaches, trainers, and strength and conditioning specialists (13.6%). Secondary sources included teammates (10.2%), family members (10.2%), and physicians (10.2%). Less frequently mentioned sources were advertising (3.4%), self-recommendation (3.4%), and specialized magazines (1.7%).

Regarding points of purchase, the most frequent sources were dietitians-nutritionists (23.7%), friends (18.6%), coaches/trainers (13.6%), and the internet (13.6%). Secondary purchase points included shopping centers (8.5%), gyms (6.8%), herbal shops (6.8%), and supermarkets (3.4%). Pharmacies (3.4%) and specialized stores (1.7%) were the least common sources.

Table 5 summarizes the ten most popular sports supplements, showing their overall usage and distribution across playing positions.

Table 6 presents SS consumption by AIS category both in total and by playing position. Based on the AIS classification [51], among the 59 players who consumed supplements, the mean number of SS was 3.1 ± 2.2, with position-specific means of 3.0 ± 1.9 for forwards, 3.7 ± 3.0 for defenders, and 2.6 ± 1.3 for midfielders, with no significant differences between positions.

Overall, there were significant differences in supplement consumption by AIS category (*p* < 0.001; η^2^ = 0.343), with Group A consumption significantly higher than both Group B (*p* < 0.001) and Group C (*p* < 0.001). No significant differences were found between Groups B and C (*p* = 0.490).

When SS consumption was analyzed by playing position, significant differences (*p* = 0.027; η^2^ = 0.073) were observed in Group C supplement use, with midfielders consuming significantly more than forwards (*p* = 0.019) and defenders (*p* = 0.021).

### 3.4. Dietary Intake and Low Energy Availability Risk

**Figure 5:**
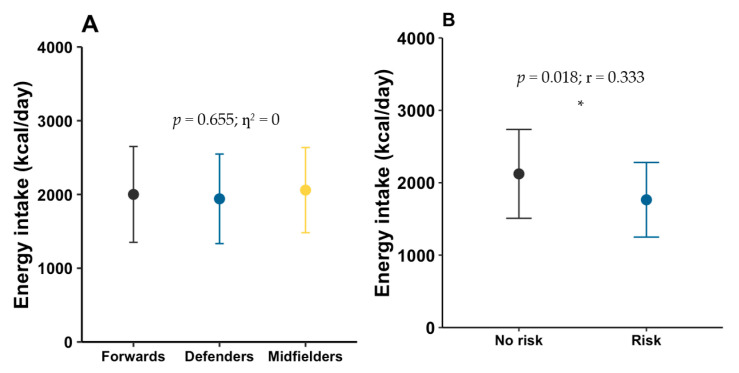
Daily energy intake by playing position (**A**) and by risk of low energy availability (**B**). * indicates statistically significant differences (*p* < 0.05).

Mean daily energy intake was 2003.1 ± 603.4 kcal/day. Mean macronutrient intake was 75.1 ± 32.5 g/day of fat, 222.7 ± 86.2 g/day of carbohydrate, and 109.0 ± 40.0 g/day of protein, with a mean fiber intake of 23.6 ± 8.0 g/day. Figure 5A shows energy intake by playing position.

**Figure 6:**
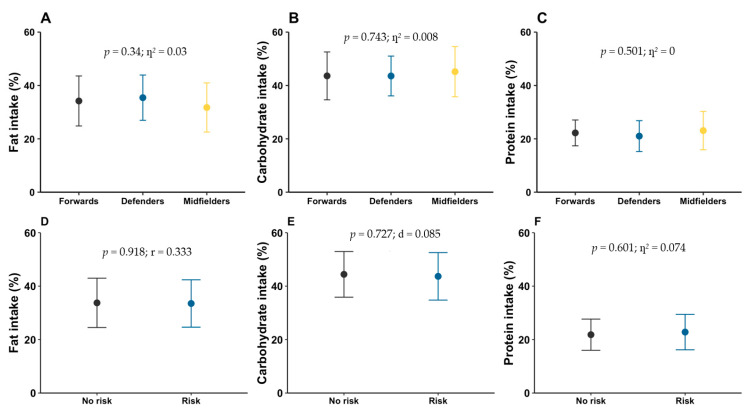
Average percentage of consumption of macronutrients by playing position (**A**–**C**) and according to LEA risk (**D**–**F**).

The percentage contribution of each macronutrient to total energy intake was, on average, 33.7 ± 9.0% from fat, 44.2 ± 8.7% from carbohydrate, and 22.2 ± 6.1% from protein. Figure 6A–C presents the macronutrient distribution by playing position.

When expressed relative to body weight, mean daily intakes were 31.9 ± 10.8 kcal/kg for energy, 1.2 ± 0.6 g/kg for fat, 3.6 ± 1.5 g/kg for carbohydrate, and 1.7 ± 0.6 g/kg for protein. Table 7 details intake by playing position.

LEAF-Q results indicated that 33.3% of the players were at risk of LEA. The mean scores were 1.3 ± 1.8 points for the injury section, 2.7 ± 2.1 points for gastrointestinal function, and 2.5 ± 2.7 points for menstrual function, yielding a total mean score of 6.5 ± 4.0 points. Section-specific and total scores by position are presented in Table 8, and the percentage distribution of LEA risk by position is presented in Table 9.

When dietary intake was compared between players at risk of LEA and those not at risk, significant differences were found only in energy intake (*p* = 0.018; η^2^ = 0.333) (Figure 5B). Figure 6D–F shows the percentage macronutrient distribution by LEA risk status.

Of the 75 athletes evaluated, 57.3% did not meet the energy intake recommendations proposed by the FIH, 34.7% met them, and 8.0% exceeded them. Considering energy intake relative to body weight (kcal/kg), 21.3% met the recommendations, while 78.7% had a reduced energy intake.

Regarding fat intake, 41.3% met recommendations, 1.3% were below, and 57.3% exceeded the recommendations relative to body weight. In percentage terms, 1.3% failed to meet the minimum, 33.3% had adequate intake, and 65.3% exceeded the recommended proportion of fat.

For protein intake relative to body weight, 9.3% were below the recommended level, 65.3% met recommendations, and 25.3% consumed excessive amounts. When expressed as a percentage of total energy intake, 16.0% consumed insufficient protein, and 84.0% met the recommended percentage.

Carbohydrate intake presented the highest rate of non-compliance, with 86.7% failing to meet recommendations relative to body weight and 98.7% failing to meet percentage-based recommendations. Only 13.3% and 1.3%, respectively, met the corresponding recommendations. Fiber intake was inadequate in 78.7% of players, while 21.3% consumed an adequate amount.

**Figure 7:**
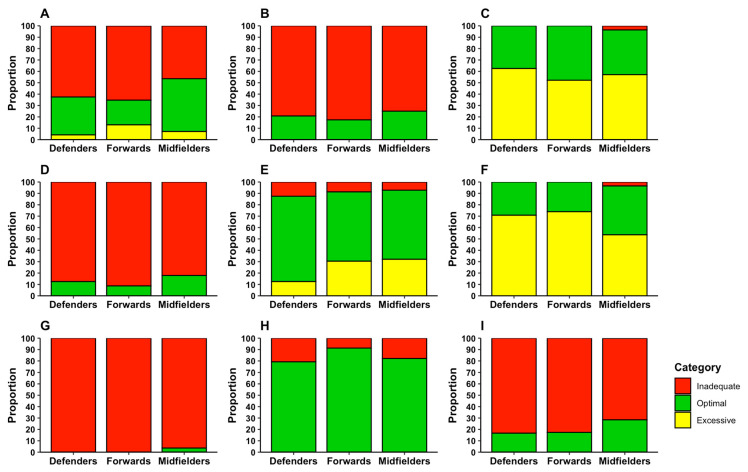
Compliance rates with dietary recommendations: energy intake according to ISSN (**A**) and FIH (**B**); fat (**C**), carbohydrates (**D**), and protein (**E**) intake per kilogram of body weight; percentage intake of fat (**F**), carbohydrates (**G**), and protein (**H**); and fiber intake compliance (**I**).

Figure 7 presents compliance with energy, macronutrient, and fiber intake recommendations by playing position. In general terms, compliance varied depending on the reference body. According to FIH values, 57.3% did not reach the recommended energy intake, 34.7% met it, and 8.0% exceeded it. However, based on ISSN recommendations, the rate of non-compliance was higher, with 78.7% failing to meet the adequate energy intake, and 21.3% achieving it.

Regarding macronutrients, patterns of adequacy varied. Carbohydrate intake was insufficient in 86.7% of athletes, with only 13.3% meeting recommendations. For protein, 65.3% met recommendations, 25.3% exceeded them, and 9.3% were below the minimum. Fat intake exceeded recommendations in 57.3% of players, met recommendations in 41.3%, and was below the minimum in 1.3%. Fiber intake was inadequate in 78.7% of players, with only 21.3% meeting recommendations.

When analyzed according to FIH recommendations, inadequacy rates were even higher. For carbohydrates, 98.7% failed to meet recommendations, with only 1.3% achieving an adequate intake. For fats, 65.3% exceeded FIH values, 33.3% met them, and 1.3% were below recommendations.

### 3.5. Low Energy Availability Risk, Sleep, Nutrition Knowledge, and Sports Supplement Relationship

Correlation analyses revealed several significant associations between the variables studied, particularly between nutrition knowledge, dietary intake, and supplement use. There was a positive correlation between the CEAC-Q score and the number of supplements used (r = 0.233; 95% confidence intervals (95% CI) 0.01 to 0.43; *p* = 0.044). Conversely, LEAF-Q scores were negatively correlated with several intake variables, notably protein intake per kilogram of body weight (r = −0.294; 95% CI −0.49 to −0.07; *p* = 0.012), percentage of protein intake (r = −0.363; 95% CI −0.55 to −0.15; *p* = 0.001), and carbohydrate intake per kilogram of body weight (r = −0.347; 95% CI −0.53 to −0.14; *p* = 0.003).

In contrast, the NUKYA questionnaire showed only weak correlations with total fat intake (r = 0.17; 95% CI −0.06 to 0.37; *p* = 0.142), fat intake relative to body weight (r = 0.18; 95% CI −0.05 to 0.38; *p* = 0.144), and energy intake relative to body weight (r = 0.09; 95% CI −0.14 to 0.31; *p* = 0.443).

## 4. Discussion

This study aimed to comprehensively evaluate the nutritional status, knowledge of sports nutrition, sleep habits, and use of SS in national-level field hockey players through an observational, cross-sectional, and descriptive study. The main findings reveal significant deficiencies among the players, including deficiencies in nutrition knowledge, sleep problems, and inadequate dietary intake—factors that may compromise the health and performance of the participants [7,55].

Nutrition knowledge, both in general and regarding the use and importance of carbohydrates in athletic performance, is essential for proper dietary planning and for optimizing sport performance [20,56], for health [22], and the acquisition of eating habits [57,58]. Despite this, some previous studies [9,59] have pointed out deficiencies in this area in female athletes, with a probable direct implication on their dietary decisions that affect their health and performance. Similarly, factors such as sleep and the use of SS can influence recovery, athletic performance, and injuries [9,60].

Although the analysis of the NUKYA questionnaire showed an average level of general nutrition knowledge (mean of 66.0 ± 8.5 points), the analysis of the CEAC-Q indicated a low level of specific knowledge about the use of carbohydrates (mean score of 24.3 ± 14.9 points). Regarding general nutrition knowledge, while comparison is difficult due to the large number and variability of existing questionnaires, these results are superior to those obtained by hockey players evaluated by Davar [25] and Vázquez-Espino et al. [57], although the latter study did not include female participants in the hockey group.

The results obtained in the questionnaire assessing general sports nutrition knowledge show a moderate level of knowledge, with an average score below the ideal threshold, as well as an insufficient level in areas such as meal planning, macronutrients, and hydration. These findings are consistent with previous studies, where it is common for athletes to have generalized deficits in their understanding of energy and macronutrient requirements or needs [59], the role of nutrients in energy metabolism [60], and even how requirements vary through the season [61]. This limited general sports nutrition knowledge is reinforced when analyzing the results regarding the use and importance of carbohydrates in sport, where more than half of the participants scored low in specific knowledge. This dual assessment provides a very comprehensive perspective and confirms that many non-professional female athletes have significant deficits in their nutritional education [59,62]. Furthermore, it has been shown that lower scores on these types of questionnaires lead to poorer food choices, which can result in nutritional deficiencies in the medium and long term [22]. It is also a general trend that athletes do not have optimal knowledge regarding current needs and recommendations for carbohydrates for competition [63].

Another relevant finding was that a significant proportion of the field hockey players evaluated had a dietary intake that did not meet the energy demands of their sport to maintain their health and performance [7,52]. The average energy consumption was 2003.1 ± 603.4 kcal/day, resulting in an average of 31.9 ± 10.8 kcal/kg of body weight, which is insufficient for athletes [49] and specifically for field hockey players [50]. It should be pointed out that media significantly shapes physical appearance standards, and the resulting pressure correlates strongly with body image concerns [64,65]. Athletes are particularly vulnerable, facing the dual demand of achieving optimal physical performance and conforming to a culturally idealized body shape [65]. This unique interaction between performance requirements and appearance focus, particularly in certain sports, can lead to tendencies toward conditions like orthorexia nervosa [65,66].

In relation to macronutrients, studies suggest a dietary pattern characterized by low carbohydrate and fiber consumption, together with a high protein and fat intake [23,26,67]. This trend is common among athletes in general [67] and more specifically among field hockey players [23,26], where Malaysian players [26] have 31.2% and 22.4% deficits in energy and carbohydrate consumption but excess consumption of 12.5% in protein and 30.6% in fat. The low amounts of carbohydrates consumed, coupled with low scores on the CEAC-Q questionnaire, corroborate the data provided by McHaffie et al. [68], which suggest that insufficient consumption of this macronutrient may be due to misconceptions regarding body composition and total body weight, together with the belief that it has a negative impact on body image, reinforced by social media, coaching staff, regular body composition monitoring, and the absence of registered dietitians-nutritionists on the team. In addition, another component with low adherence in consumption is fiber, with 78.7% of participants not reaching the minimum requirement. This, together with low carbohydrate consumption, may be indicative of poor consumption of plant-based foods such as whole grains, legumes, fruits, and vegetables [69], negatively affecting digestive health and microbiome stability [48].

Of the total number of participants, 33.3% were classified as at risk of LEA, a high and worrying percentage with physiological consequences such as menstrual dysfunction, bone loss, alterations in immune function, and reduced athletic performance [7,70,71]. A low-calorie, low-carbohydrate diet in female athletes leads to physiological changes that compromise health and performance. This state of LEA interferes with basic endocrine functions, impairing the secretion of various hormones (leptin, insulin, luteinizing hormone, and thyroid), all critical for proper metabolic and reproductive homeostasis [72,73]. This results in menstrual dysfunction, along with a reduction in basal metabolic rate, which is an adaptive strategy of the body to save energy [71,72]. At the metabolic level, limited carbohydrate intake decreases available muscle and liver glycogen, reducing the ability to withstand high-intensity training [71,73]. Furthermore, these dietary deficiencies affect bone health by reducing bone mineral density and increasing the risk of stress fractures [74,75]. At the immunological level, there is also an increase in respiratory infections and slower recovery [73,74]. These alterations do not occur in isolation but often coexist with symptoms such as fatigue, sleep disorders, anxiety, and a higher prevalence of dysfunctional eating behaviors, including voluntary restriction of carbohydrates [76]. Therefore, ensuring adequate energy availability and optimal carbohydrate consumption is a priority in sports nutrition planning for female athletes [71].

In addition to these data on physiological and metabolic consequences, more than half of the women surveyed by Tenforde et al. [77] believe that being thinner leads to running faster. Although nutritional knowledge is one of the most relevant modifiable factors in dietary intake [20], and there is a positive correlation between nutritional knowledge and dietary habits and body composition [60], research conducted by Burger et al. [19] found that female athletes with greater nutrition knowledge are more likely to be in a state of LEA (16.5 points versus 14.5 points, *p* < 0.01), results contrary to the work carried out by Pai et al. [78], in which women classified as at risk of LEA had significantly lower levels of nutrition knowledge.

The second relevant aspect to consider is the results obtained from the ASSQ questionnaire, which showed an average of mild sleep disturbances. However, 33.3% of participants presented moderate to severe sleep disturbances, and the vast majority (92.0%) sleep less than 8 h, along with widespread dissatisfaction with their daily night-time sleep, which is a common problem among the Spanish population, especially among women and people who use electronic devices, leading to an increased risk of poorer mental health [79]. These data may be justified by the high percentage (90.7%) of electronic device use prior to bedtime. This is a very common practice among team sports athletes [80]. On average, electronic devices are used for 4.6 h per day, meaning that high use of these devices has a negative impact on sleep quality and can hinder athletic performance [81] by decreasing endogenous melatonin synthesis and increasing alertness [10]. Moreover, it is important to consider factors such as sports event schedules, the athlete’s psychosocial stress, travel, the use of stimulant substances like caffeine, and alcohol intake, among others. Various strategies, including sleep extension, napping, sleep hygiene practices, and nutritional strategies, should be considered for improving athlete sleep [9,11].

Regarding supplement consumption, 78.7% of players reported using SS, with an average of 3.2 ± 2.2 SS per person, with performance being the main reason for use (37.3%). However, in most cases, they did not appear to have professional supervision, as it is the Dietitian-Nutritionist who should make specific recommendations on SS [44], helping to ensure responsible use of SS based on scientific evidence [43,44]. It has been shown that athletes who are supported by a dietitian-nutritionist tend to have better eating habits, especially in terms of nutritional periodization, and consumption of dietary supplements with positive scientific evidence in their favor [82,83,84] and to avoid fraud in the composition of SS [85]. Furthermore, it is worth noting the discrepancy found between the main sources of information on SSs (friends, internet, dietitians-nutritionists in first, second, and third place, respectively) and the main places of purchase (dietitians-nutritionist), which could indicate a possible case of professional intrusion. Although sports supplements are commonly used by athletes, their implementation is frequently suboptimal and inadequate [86,87]. Therefore, it is crucial for both health professionals and athletes to conduct a cost–benefit analysis before their use, considering their safety, efficacy, and legality [87,88].

It should therefore be noted that educational interventions in young athletes [22] can provide various benefits, such as improved dietary habits [89], improved body composition [89,90,91], and even facilitation of recovery from relative energy deficiency syndrome in sport [92]. Despite these benefits, there is no fixed intervention [91], so this type of intervention should be planned and carried out according to the collective need of each sports team, addressing the identified deficiencies in nutritional knowledge, inadequate dietary patterns, and potential risk of LEA.

### Limitations and Future Research

Although the results obtained may be relevant, this study has certain limitations that must be considered. First, the cross-sectional approach cannot determine causality. As this is an observational study, significant associations do not imply causation. Effect sizes and confidence intervals were emphasized to assess practical significance and avoid overinterpreting minor effects. In addition, the use of self-administered questionnaires may lead to memory bias [93] or social desirability bias [94]. Another major limitation in this type of research is the wide variability that exists among questionnaires that attempt to determine the level of nutrition knowledge among athletes [59].

On the other hand, although the sample size is adequate, the conclusions cannot be extrapolated to all field hockey players or other disciplines due to differences in training load and distribution, competitive level, and sociocultural context. Another limitation of this study was that the sampling was non-probabilistic and based on convenience, as the questionnaire was sent to all players in the Honor B Division League, but only 75 ultimately participated. Finally, the assessment of dietary intake using a 7-day record may underestimate dietary intake due to recording errors or significant changes in behavior during the observation period [95].

Additionally, future research should aim to complement questionnaire-based screening with direct measurements of energy availability, including exercise energy expenditure and fat-free mass calculations, as well as physiological markers, to provide more accurate and comprehensive EA measurements.

## 5. Conclusions

The results of this study show that, despite demonstrating a high level of general nutrition knowledge, female field hockey players have marked deficiencies in specific knowledge about the use of carbohydrates. Dietary intake was insufficient in energy and, more specifically, in carbohydrates and fiber, leading to the conclusion that 33.3% of the participants are at risk of low energy availability. In addition, more than 60% of the female players had sleep disturbances, which can negatively affect recovery. The use of sports supplements is high, although many resort to non-professional sources for their consumption. These findings highlight the need for specific educational and nutritional interventions, tailored to the needs of female athletes, considering rest and nutrition in a comprehensive manner.

## Figures and Tables

**Table 1:** Energy and macronutrient recommendations for field hockey athletes.

Dietary Component	Recommendation	Unity	Reference
Energy	40 to 70	kcal/kg/BW	[49]
	2000 to 3000	Kcal/day	[50]
Carbohydrates	5 to 10	g/kg/BW	[49,51,52]
	60 to 60	% of energy	[50]
Protein	1 to 2	g/kg/BW	[49,50,51,52]
Fats	0.5 to 1	g/kg/BW	[49]
	15 to 30	% of energy	[50]

BW, body weight.

**Table 2:** Demographics characteristics of participants.

Variables	Overall (*n* = 75)	Forwards (*n* = 23)	Defenders (*n* = 24)	Midfielders (*n* = 28)	*p*	η^2^
Age (years)	24.0 ± 5.0	20.7 ± 4.9	24.4 ± 5.0	24.7 ± 5.2	0.275	0.000
Height (cm)	165.6 ± 6.0	164.8 ± 4.6	167.0 ± 5.0	165.1 ± 7.5	0.407	0.025
Body Weight (kg)	64.1 ± 9.2	64.1 ± 9.9	64.5 ± 9.2	63.7 ± 8.9	0.940	0.000
BMI (kg/m^2^)	23.3 ± 2.5	23.5 ± 2.7	23.1 ± 2.8	23.3 ± 2.1	0.158	0.004
Hockey experience (years)	8.7 ± 2.9	8.7 ± 3.1	9.1 ± 2.8	8.3 ± 2.8	0.522	0.000
Training frequency (days/week)	5.8 ± 2.2	5.6 ± 1.9	5.8 ± 2.2	5.9 ± 2.4	0.893	0.000
Training volume (hours/week)	10.5 ± 5.4	9.6 ± 4.8	10.7 ± 5.9	11.1 ± 5.6	0.484	0.000
Hockey training frequency (days/week)	3.9 ± 1.4	3.7 ± 1.3	3.8 ± 1.5	4.0 ± 1.4	0.840	0.005
Hockey training volume (hours/week)	8.3 ± 4.2	7.6 ± 3.6	8.4 ± 4.5	8.9 ± 4.5	0.612	0.00
Complementary training frequency (days/week)	1.9 ± 1.3	1.8 ± 1.2	2.0 ± 1.3	1.9 ± 1.4	0.934	0.002
Complementary training volume (hours/week)	2.2 ± 1.9	2.0 ± 1.8	2.4 ± 2.1	2.2 ± 1.9	0.671	0.000

Data are represented as means and standard deviations, unless otherwise indicated. Height was self-reported. BMI, Body Mass Index.

**Table 3:** Percentage distribution of sleep disturbances among female hockey players by playing position.

SDS Category	Forwards (*n* = 23)	Defenders (*n* = 24)	Midfielders (*n* = 28)
None	26.1	16.7	32.1
Mild	34.8	45.8	42.9
Moderate	21.7	33.3	10.7
Severe	17.4	4.2	14.3

Data are represented as percentages.

**Table 4:** Responses by section of the ASSQ questionnaire by playing position.

ASSQ Section	ASSQ Item	Forwards (*n* = 23)	Defenders (*n* = 24)	Midfielders (*n* = 28)
Sleep nighttime	5 to 6 h	39.1	37.5	28.6
	6 to 7 h	30.5	25.0	32.1
	7 to 8 h	26.1	20.1	35.7
	8 to 9 h	4.3	16.7	3.6
	More than 9 h	0.0	0.0	0.0
Sleep satisfaction	Very satisfied	26.1	16.7	21.4
	Somewhat satisfied	21.7	16.7	35.7
	Neither satisfied nor dissatisfied	17.4	45.8	14.3
	Somewhat dissatisfied	30.4	20.8	28.6
	Very dissatisfied	4.3	0.0	0.0
Difficulty falling sleep	None	30.4	41.7	32.1
	Once or twice per week	52.2	45.8	50.0
	Three or four times per week	13.0	20.8	7.1
	Five to seven days per week	4.3	0.0	10.7
Use of electronic devices	Not at all	4.3	4.2	17.9
	1 to 3 times per week	43.5	41.7	35.7
	4 to 6 times per week	4.3	8.3	14.3
	Every day	73.9	70.8	60.7
	Not at all	4.3	4.2	17.9

Data are represented as percentages.

**Table 5:** Top ten most frequently consumed sport supplements in the total sample and by playing position.

Total (*n* = 59)		Forwards (*n* = 20)		Defenders (*n* = 19)		Midfielders (*n* = 20)	
Supplement	*n* (%)	Supplement	*n* (%)	Supplement	*n* (%)	Supplement	*n* (%)
Caffeine	17 (28.8)	Caffeine	8 (40.0)	Vitamin D	6 (31.6)	Creatine	6 (30.0)
Creatine	16 (27.1)	Creatine	6 (30.0)	Iron	4 (21.1)	Whey Protein	5 (25.0)
Isotonic Drink	12 (20.3)	Vitamin C	5 (25.0)	Vitamin C	4 (21.1)	Caffeine	5 (25.0)
Protein Bar	11 (18.6)	Electrolytes	4 (20.0)	Creatine	4 (21.1)	Isotonic Drink	5 (25.0)
Vitamin C	11 (18.6)	Isotonic Drink	4 (20.0)	Omega 3	4 (21.1)	Multivitamin	5 (25.0)
Whey Protein	10 (16.9)	Magnesium	4 (20.0)	Protein Bar	4 (21.1)	Omega 3	4 (20.0)
Omega 3	10 (16.9)	Iron	3 (15.0)	Caffeine	4 (21.1)	Protein Bar	4 (20.0)
Vitamin D	9 (15.3)	Sport Bar	3 (15.0)	Whey Protein	3 (15.8)	Sport Bar	3 (15.0)
Sport Bar	9 (15.3)	Protein Bar	3 (15.0)	Protein Bar	3 (15.8)	Vitamin C	2 (10.0)
Iron	8 (13.6)	Omega 3	2 (10.0)	Isotonic Drink	3 (15.8)	Vitamin D	2 (10.0)

**Table 6:** Number of sports supplements consumed depending on playing position according to AIS category.

AIS Category		Overall (*n* = 75)	Forwards (*n* = 23)	Defenders (*n* = 24)	Midfielders (*n* = 28)	*p*	η^2^
Group A	Sports Foods	1.0 ± 1.1	0.9 ± 1.0	1.1 ± 1.2	1.0 ± 1.3	0.886	0.000
	Medical Supplements	0.4 ± 0.7	0.3 ± 0.6	0.6 ± 0.9	0.4 ± 0.7	0.443	0.000
	Ergogenic Aids	0.6 ± 0.6	0.8 ± 0.7	0.6 ± 0.7	0.6 ± 0.6	0.699	0.000
	Total Group A	2.1 ± 1.4	2.0 ± 1.4	2.3 ± 1.7	2.0 ± 1.1	0.678	0.000
Group B		0.4 ± 0.7	0.4 ± 0.6	0.5 ± 0.8	0.4 ± 0.7	0.870	0.000
Group C		0.6 ± 1.0	0.7 ± 0.8	0.9 ± 1.3	0.2 ± 0.5	0.027	0.073

Data are represented as means and standard deviations, unless otherwise indicated.

**Table 7:** Dietary intake by playing position.

Variables	Forwards (*n* = 23)	Defenders (*n* = 24)	Midfielders (*n* = 28)	*p*	η^2^
Energy (kcal/day)	2000.4 ± 649.7	1940.7 ± 607.0	2058.8 ± 577.5	0.777	0.000
Energy (kcal/kg BW/day)	32.4 ± 12.0	30.1 ± 9.3	33.7 ± 10.8	0.709	0.000
Carbohydrates (g/day)	220.4 ± 75.9	214.2 ± 82.7	231.8 ± 98.3	0.759	0.000
Carbohydrates (kcal/kg BW/day)	3.5 ± 1.5	3.4 ± 1.4	3.7 ± 1.6	0.619	0.000
Protein (g/day)	112.0 ± 36.7	103.3 ± 42.0	111.6 ± 38.6	0.196	0.018
Protein (kcal/kg BW/day)	1.8 ± 0.6	1.6 ± 0.6	1.8 ± 0.6	0.232	0.000
Fats (g/day)	74.1 ± 36.7	70.5 ± 22.3	79.8 ± 36.5	0.966	0.000
Fats (kcal/kg BW/day)	1.2 ± 0.7	1.1 ± 0.4	1.3 ± 0.6	0.948	0.000
Fiber (g/day)	23.5 ± 7.7	21.5 ± 8.3	25.4 ± 7.7	0.386	0.000

Data are represented as means and standard deviations, unless otherwise indicated.

**Table 8:** Total and section scores obtained by playing position in the LEAF-Q questionnaire.

Section	Forwards (*n* = 23)	Defenders (*n* = 24)	Midfielders (*n* = 28)	*p*	η^2^
Injuries	1.1 ± 1.8	0.8 ± 1.5	1.9 ± 2.0	0.080	0.068
Gastrointestinal function	2.7 ± 2.0	2.7 ± 2.4	2.7 ± 2.1	0.999	0.000
Menstrual function	2.6 ± 3.0	2.4 ± 2.2	2.5 ± 2.0	0.963	0.001
Total	6.4 ± 4.5	5.9 ± 4.5	7.1 ± 3.0	0.518	0.015

Data are represented as means and standard deviations, unless otherwise indicated.

**Table 9:** LEAF-Q Scores by playing position.

Position	LEAF-Q < 8 Points (No Risk)	LEAF-Q > 8 Points (Risk)
Forwards (*n* = 23)	69.6%	30.4%
Defenders (*n* = 24)	70.8%	29.2%
Midfielders (*n* = 28)	60.7%	39.3%

## Data Availability

In alignment with Research Data Policies promoting open science, the dataset from this study is accessible to interested researchers via the Figshare repository: https://doi.org/10.6084/m9.figshare.30893156, accessed on 15 November 2025.

## Supplementary Materials

The following supporting information can be downloaded at: https://www.mdpi.com/article/10.3390/nu17243934/s1, Table S1. Frequencies of reason for the use of sports supplements; Table S2. Frequencies of recommendation for the use of sports supplementation; Table S3. Frequencies of place of purchase of sports supplements; Table S4. Frequencies of sports supplement intake by time of day; Table S5. Frequencies of sports supplement intake timing; Table S6. Frequencies of supplement consumption according to the AIS classification; Table S7. ASSQ Question: During the recent past, how many hours of actual sleep did you get at night? (This may be different than the number of hours you spent in bed); Table S8. ASSQ Question: How satisfied/dissatisfied are you with the quality of your sleep?; Table S9. ASSQ Question: During the recent past, how long has it usually taken you to fall asleep each night?; Table S10. ASSQ Question: How often do you have trouble staying asleep?; Table S11. ASSQ Question: During the recent past, how often have you taken medicine to help you sleep (prescribed or over-the-counter)?; Table S12. ASSQ Question: Considering only your own “feeling best” rhythm, at what time would you get up if you were entirely free to plan your day?; Table S13. ASSQ Question: Do you consider yourself to be a morning type person or an evening type person?; Table S14. ASSQ Question: Considering your own “feeling best” rhythm, at what time would you go to bed if you were entirely free to plan your evening?; Table S15. ASSQ Question: When you are travelling for your sport, do you experience sleep disturbance?; Table S16. ASSQ Question: When you are travelling for your sport, do you experience daytime dysfunction (feeling generally unwell or having poor performance)?; Table S17. ASSQ Question: Are you typically a loud snorer?; Table S18. ASSQ Question: Have you been told that you choke, gasp, or stop breathing for periods of time during sleep?; Table S19. ASSQ Question: On average, how many caffeinated products (caffeine pills, coffee, tea, soda, energy drinks) do you have per day? For coffee and tea, one drink = 6–8 oz/177–237 mL; for caffeinated soda, one drink = 1 can (12 oz/355 mL)?; Table S20. ASSQ Question: Over the recent past, how often do you use an electronic device (example: cell phone, computer, tablet, T.V. etc.) within 1 h of going to bed?.

## Abbreviations

The following abbreviations are used in this manuscript:

AIS	Australian Institute of Sports
ASSQ	Athletes Sleep Screening Questionnaire
CEAC-Q	Carbohydrate for Endurance Athletes in Competition Questionnaire
ES	Effect Size
LEA	Low Energy Availability
LEAF-Q	Low Energy Availability in Females Questionnaire
NUKYA	Nutrition Knowledge for Young and Adult Athletes
SD	Standard Deviation
SS	Sports Supplements
