# Supplementary material for: Knowledge Gaps, Sleep Disturbances, and Energy Imbalance Among Female Field Hockey Players
Source: Nutrients. 2025 Dec 16;17(24):3934. doi: 10.3390/nu17243934 (PMC12735660; doi:10.3390/nu17243934)
Supplement: Supplementary file 1 [file nutrients-17-03934-s001.zip › nutrients-3984995-supplementary.pdf]

Table S1. Frequencies of reason for the use of sports supplements.

Reason	Overall (n = 59)	Forwards (n = 20)	Defenders (n = 19)	Midfielders (n = 20)
Sports performance	28 (47.5 %)	8 (40.0 %)	10 (52.6 %)	10 (50.0 %)
Health maintenance	17 (28.8 %)	5 (25.0 %)	6 (31.6 %)	6 (30.0 %)
Improving physical appearance	5 (8.5 %)	2 (10.0 %)	1 (5.3 %)	2 (10.0 %)
Compensating for dietary deficiencies	4 (6.8 %)	3 (15.0 %)	1 (5.3 %)	0 (0.0 %)
Addressing health problems	2 (3.4 %)	2 (10.0 %)	0 (0.0 %)	0 (0.0 %)
Necessity	1 (1.7 %)	0 (0.0 %)	1 (5.3 %)	0 (0.0 %)
Habit	1 (1.7 %)	0 (0.0 %)	0 (0.0 %)	1 (5.0 %)
Obligation	1 (1.7 %)	0 (0.0 %)	0 (0.0 %)	1 (5.0 %)

Data is presented as n (%).

Table S2. Frequencies of recommendation for the use of sports supplementation.

Recommended by	Overall (n = 59)	Forwards (n = 20)	Defenders (n = 19)	Midfielders (n = 20)
Friends	11 (18.6 %)	5 (25.0 %)	4 (21.1 %)	2 (10.0 %)
Internet	9 (15.3 %)	4 (20.0 %)	1 (5.3 %)	4 (20.0 %)
Dietitian-Nutritionist	8 (13.6 %)	1 (5.0 %)	3 (15.8 %)	4 (20.0 %)
Coaches, trainers, and strength and conditioning specialist	8 (13.6 %)	3 (15.0 %)	3 (15.8 %)	2 (10.0 %)
Teammates	6 (10.2 %)	2 (10.0 %)	1 (5.3 %)	3 (15.0 %)
Family members	6 (10.2 %)	2 (10.0 %)	2 (10.5 %)	2 (10.0 %)
Physicians	6 (10.2 %)	1 (5.0 %)	3 (15.8 %)	2 (10.0 %)
Advertising	2 (3.4 %)	1 (5.0 %)	1 (5.3 %)	0 (0.0 %)
Self-recommendation	2 (3.4 %)	1 (5.0 %)	0 (0.0 %)	1 (5.0 %)
Specialized magazines	1 (1.7 %)	0 (0.0 %)	1 (5.3 %)	0 (0.0 %)

Data is presented as n (%).

Table S3. Frequencies of place of purchase of sports supplements.

Place of purchase	Overall (n = 59)	Forwards (n = 20)	Defenders (n = 19)	Midfielders (n = 20)
Dietitian-Nutritionist	14 (23.7 %)	6 (30.0 %)	1 (5.3 %)	7 (35.0 %)
Friends	11 (18.6 %)	3 (15.0 %)	5 (26.3 %)	3 (15.0 %)
Coaches/trainers	8 (13.6 %)	4 (20.0 %)	1 (5.3 %)	3 (15.0 %)
Internet	8 (13.6 %)	1 (5.0 %)	4 (21.1 %)	3 (15.0 %)
Shopping center	5 (8.5 %)	1 (5.0 %)	2 (10.5 %)	2 (10.0 %)
Gyms	4 (6.8 %)	1 (5.0 %)	2 (10.5 %)	1 (5.0 %)
Herbal shops	4 (6.8 %)	2 (10.0 %)	1 (5.3 %)	1 (5.0 %)
Supermarkets	2 (3.4 %)	1 (5.0 %)	1 (5.3 %)	0 (0.0 %)
Pharmacies	2 (3.4 %)	0 (0.0 %)	2 (10.5 %)	0 (0.0 %)
Specialized stores	1 (1.7 %)	1 (5.0 %)	0 (0.0 %)	0 (0.0 %)

Data is presented as n (%).

Table S4. Frequencies of sports supplement intake by time of day.

Moment of the day	Overall (n = 59)	Forwards (n = 20)	Defenders (n = 19)	Midfielders (n = 20)
In competitions	11 (18.6 %)	2 (10.0 %)	4 (21.1 %)	5 (25.0 %)
In trainings	7 (11.9 %)	4 (20.0 %)	1 (5.3 %)	2 (10.0 %)
In trainings and competitions	19 (32.2 %)	8 (40.0 %)	5 (26.3 %)	6 (30.0 %)
Indifferent	14 (23.7 %)	4 (20.0 %)	6 (31.6 %)	4 (20.0 %)
During holidays or rest periods	4 (6.8 %)	2 (10.0 %)	2 (10.5 %)	0 (0.0 %)
In the mornings	1 (1.7 %)	0 (0.0 %)	0 (0.0 %)	1 (5.0 %)
Every day	3 (5.1 %)	0 (0.0 %)	2 (10.5 %)	1 (5.0 %)

Data is presented as n (%).

Table S5. Frequencies of sports supplement intake timing.

Timing	Overall (n = 59)	Forwards (n = 20)	Defenders (n = 19)	Midfielders (n = 20)
Before practice	25 (42.4 %)	7 (35.0 %)	8 (42.1 %)	10 (50.0 %)
During practice	8 (13.6 %)	3 (15.0 %)	2 (10.5 %)	3 (15.0 %)
After practice	15 (25.4 %)	7 (35.0 %)	6 (31.6 %)	2 (10.0 %)
Indifferent	11 (18.6 %)	3 (15.0 %)	3 (15.8 %)	5 (25.0 %)

Data is presented as n (%).

Table S6. Frequencies of supplement consumption according to the AIS classification.

	AIS Category	Overall (n = 59)	Forwards (n = 20)	Defenders (n = 19)	Midfielders (n = 20)
Group A	Sports Foods	35 (59.3 %)	11 (55.0 %)	12 (63.2 %)	12 (60.0 %)
	Medical Supplements	19 (32.2 %)	5 (25.0 %)	8 (42.1 %)	5 (25.0 %)
	Ergogenic Aids	32 (54.2 %)	12 (60.0 %)	9 (47.4 %)	11 (55.0 %)
	Total Group A	53 (89.8 %)	19 (95.0 %)	16 (84.2 %)	18 (90.0 %)
	Group B	20 (33.9 %)	7 (35.0 %)	7 (36.8 %)	6 (30.0 %)
	Group C	20 (33.9 %)	10 (50.0 %)	8 (42.1 %)	2 (10.0 %)

Data is presented as n (%).

Table S7. ASSQ Question: During the recent past, how many hours of actual sleep did you get at night? (This may be different than the number of hours you spent in bed.)

Response	Overall (n = 75)	Forwards (n = 23)	Defenders (n = 24)	Midfielders (n = 28)
5 to 6 hours	26 (34.7 %)	9 (39.1 %)	9 (37.5 5)	8 (28.6 %)
6 to 7 hours	22 (29.3 %)	7 (30.4 %)	6 (25.0 %)	9 (32.1 %)
7 to 8 hours	21 (28.0 %)	6 (26.1 %)	5 (20.8 %)	10 (35.7 %)
8 to 9 hours	6 (8.0 %)	1 (4.3 %)	4 (16.7 %)	1 (3.6 %)
More than 9 hours	0 (0.0 %)	0 (0.0 %)	0 (0.0 %)	0 (0.0 %)

Data is presented as n (%).

Table S8. ASSQ Question: How satisfied/dissatisfied are you with the quality of your sleep?

Response	Overall (n = 75)	Forwards (n = 23)	Defenders (n = 24)	Midfielders (n = 28)
Very satisfied	16 (21.3 %)	6 (26.1 %)	4 (16.7 %)	6 (21.4 %)
Somewhat satisfied	19 (25.3 %)	5 (21.7 %)	4 (16.7 %)	10 (35.7 %)
Neither satisfied nor dissatisfied	19 (25.3 %)	4 (17.4 %)	11 (45.8 %)	4 (14.3 %)
Somewhat dissatisfied	20 (26.7 %)	7 (30.4 %)	5 (20.8 %)	8 (28.6 %)
Very dissatisfied	1 (1.3 %)	1 (4.3 %)	0 (0.0 %)	0 (0.0 %)

Data is presented as n (%).

Table S9. ASSQ Question: During the recent past, how long has it usually taken you to fall asleep each night?

Response	Overall (n = 75)	Forwards (n = 23)	Defenders (n = 24)	Midfielders (n = 28)
15 minutes or less	33 (44.0 %)	10 (43.5 %)	10 (41.7 %)	13 (46.4 %)
16 – 30 minutes	25 (33.3 %)	7 (30.4 %)	8 (33.3 %)	10 (35.7 %)
31 – 60 minutes	8 (10.7 %)	1 (4.3 %)	5 (20.8 %)	2 (7.1 %)
Longer than 60 minutes	9 (12.0 %)	5 (21.7 %)	1 (4.2 %)	3 (10.7 %)

Data is presented as n (%).

Table S10. ASSQ Question: How often do you have trouble staying asleep?

Response	Overall (n = 75)	Forwards (n = 23)	Defenders (n = 24)	Midfielders (n = 28)
None	26 (34.7 %)	7 (30.4 %)	10 (41.7 %)	9 (32.1 %)
Once or twice per week	37 (36.0 %)	12 (52.2 %)	11 (45.8 %)	14 (50.0 %)
Three or four times per week	8 (10.7)	3 (13.0 %)	3 (12.5 %)	2 (7.1 %)
Five to seven days per week	4 (5.3)	1 (4.3 %)	0 (0.0 %)	3 (10.7 %)

Data is presented as n (%).

Table S11. ASSQ Question: During the recent past, how often have you taken medicine to help you sleep (prescribed or over-the-counter)?

Response	Overall (n = 75)	Forwards (n = 23)	Defenders (n = 24)	Midfielders (n = 28)
None	67 (89.3 %)	22 (95.7 %)	20 (83.3 %)	25 (89.3 %)
Once or twice per week	3 (4.0 %)	0 (0.0 %)	3 (12.5 %)	0 (0.0 %)
Three or four times per week	3 (4.0 %)	1 (4.3 %)	1 (4.2 %)	1 (3.6 %)
Five to seven times per week	2 (2.7 %)	0 (0.0 %)	0 (0.0 %)	2 (7.1 %)

Data is presented as n (%).

Table S12. ASSQ Question: Considering only your own "feeling best" rhythm, at what time would you get up if you were entirely free to plan your day?

Response	Overall (n = 75)	Forwards (n = 23)	Defenders (n = 24)	Midfielders (n = 28)
5:00 am – 6:30 am	3 (4.0 %)	1 (4.3 %)	0 (0.0 %)	2 (7.1 %)
6:30 am – 7:45 am	26 (34.7 %)	11 (47.8 %)	7 (29.2 %)	8 (28.6 %)
7:45 am – 9:45 am	31 (41.3 %)	7 (30.4 %)	10 (41.7 %)	14 (50.0 %)
9:45 am – 11:00 am	15 (20.0 %)	4 (17.4 %)	7 (29.2 %)	4 (14.3 %)
11:00 am – 12:00 pm (noon)	0 (0.0 %)	0 (0.0 %)	0 (0.0 %)	0 (0.0 %)

Data is presented as n (%).

Table S13. ASSQ Question: Do you consider yourself to be a morning type person or an evening type person?

Response	Overall (n = 75)	Forwards (n = 23)	Defenders (n = 24)	Midfielders (n = 28)
Definitely a morning type	16 (21.3 %)	4 (17.4 %)	4 (16.7 %)	8 (28.6 %)
More a morning type than an evening type	29 (38.7 %)	8 (34.8 %)	11 (45.8 %)	10 (35.7 %)
More an evening type than a morning type	24 (32.0 %)	10 (43.5 %)	6 (25.0 %)	8 (28.6 %)
Definitely an evening type	6 (8.0 %)	1 (4.3 %)	3 (12.5 %)	2 (7.1 %)

Data is presented as n (%).

Table S14. ASSQ Question: Considering your own "feeling best" rhythm, at what time would you go to bed if you were entirely free to plan your evening?

Response	Overall (n = 75)	Forwards (n = 23)	Defenders (n = 24)	Midfielders (n = 28)
8:00 pm – 9:00 pm	5 (6.7 %)	2 (8.7 %)	2 (8.3 %)	1 (3.6 %)
9:00 pm – 10:15 pm	7 (9.3 %)	3 (13.0 %)	2 (8.3 %)	2 (7.1 %)
10:15 pm – 12:30 am	51 (68.0 %)	13 (56.5 %)	17 (70.8 %)	21 (75.0 %)
12:30 am – 1:45 am	11 (14.7 %)	5 (21.7 %)	2 (8.3 %)	4 (14.3 %)
1:45 am – 3:00 am	1 (1.3 %)	0 (0.0 %)	1 (4.2 %)	0 (0.0 %)

Data is presented as n (%).

Table S15. ASSQ Question: When you are travelling for your sport, do you experience sleep disturbance?

Response	Overall (n = 75)	Forwards (n = 23)	Defenders (n = 24)	Midfielders (n = 28)
Yes	15 (20.0 %)	3 (13.0 %)	5 (20.8 %)	7 (25.0 %)
No	60 (80.0 %)	20 (87.0 %)	19 (79.2 %)	21 (75.0 %)

Data is presented as n (%).

Table S16. ASSQ Question: When you are travelling for your sport, do you experience daytime dysfunction (feeling generally unwell or having poor performance)?

Response	Overall (n = 75)	Forwards (n = 23)	Defenders (n = 24)	Midfielders (n = 28)
Yes	20 (26.7 %)	6 (26.1 %)	7 (29.2 %)	7 (25.0 %)
No	55 (73.3 %)	17 (73.9 %)	17 (70.8 %)	21 (75.0 %)

Data is presented as n (%).

Table S17. ASSQ Question: Are you typically a loud snorer?

Response	Overall (n = 75)	Forwards (n = 23)	Defenders (n = 24)	Midfielders (n = 28)
Yes	7 (9.3 %)	2 (8.7 %)	1 (4.2 %)	4 (14.3 %)
No	68 (90.7 %)	21 (91.3 %)	23 (95.8 %)	24 (85.7 %)

Data is presented as n (%).

Table S18. ASSQ Question: Have you been told that you choke, gasp, or stop breathing for periods of time during sleep?

Response	Overall (n = 75)	Forwards (n = 23)	Defenders (n = 24)	Midfielders (n = 28)
Yes	3 (4.0 %)	1 (4.3 %)	1 (4.2 %)	1 (3.6 %)
No	72 (96.0 %)	22 (95.7 %)	23 (95.8 %)	27 (96.4 %)

Data is presented as n (%).

Table S19. ASSQ Question: On average, how many caffeinated products (caffeine pills, coffee, tea, soda, energy drinks) do you have per day? For coffee and tea, one drink = 6-8oz/177- 237ml; for caffeinated soda, one drink = 1 can (12oz/355ml)?

Response	Overall (n = 75)	Forwards (n = 23)	Defenders (n = 24)	Midfielders (n = 28)
Less than 1 per day	35 (46.7 %)	11 (47.8 %)	12 (50.0 %)	12 (42.9 %)
1-2 per day	33 (44.0 %)	10 (43.5 %)	10 (41.7 %)	13 (46.4 %)
3 per day	5 (6.7 %)	1 (4.3 %)	2 (8.3 %)	2 (7.1 %)
4 per day	2 (2.7 %)	1 (4.3 %)	0 (0.0 %)	1 (3.6 %)
5 or more per day	0 (0.0 %)	0 (0.0 %)	0 (0.0 %)	0 (0.0 %)

Data is presented as n (%).

Table S20. ASSQ Question: Over the recent past, how often do you use an electronic device (example: cell phone, computer, tablet, T.V. etc.) within 1 hour of going to bed?

Response	Overall (n = 75)	Forwards (n = 23)	Defenders (n = 24)	Midfielders (n = 28)
Not at all	7 (9.3 %)	1 (4.3 %)	1 (4.2 %)	5 (17.9 %)
1-3 times per week	10 (13.3 %)	4 (17.4 %)	4 (16.7 %)	2 (7.1 %)
4-6 times per week	7 (9.3 %)	1 (4.3 %)	2 (8.3 %)	4 (14.3 %)
Every day	51 (68.0 %)	17 (73.9 %)	17 (70.8 %)	17 (60.7 %)

Data is presented as n (%).
